# Giant field-like torque by the out-of-plane magnetic spin Hall effect in a topological antiferromagnet

**DOI:** 10.1038/s41467-021-26453-y

**Published:** 2021-11-18

**Authors:** Kouta Kondou, Hua Chen, Takahiro Tomita, Muhammad Ikhlas, Tomoya Higo, Allan H. MacDonald, Satoru Nakatsuji, YoshiChika Otani

**Affiliations:** 1 RIKEN, Center for Emergent Matter Science (CEMS), Saitama, 351-0198 Japan; 2 CREST, Japan Science and Technology Agency (JST), Kawaguchi, Saitama, 332-0012 Japan; 3 Department of Physics, Colorado State University, Fort Collins, CO USA; 4 School of Advanced Materials Discovery, Colorado State University, Fort Collins, CO USA; 5 Institute for Solid State Physics, The University of Tokyo, Kashiwa, Chiba, 277-8581 Japan; 6 Department of Physics, University of Tokyo, Hongo, Bunkyo-ku, Tokyo, 113-0033 Japan; 7 Department of Physics, University of Texas at Austin, Austin, TX USA; 8 Trans-scale Quantum Science Institute, University of Tokyo, Tokyo, Japan; 9 Institute for Quantum Matter and Department of Physics and Astronomy, Johns Hopkins University, Baltimore, MD 21218 USA

**Keywords:** Magnetic properties and materials, Topological insulators, Spintronics

## Abstract

Spin-orbit torques (SOT) enable efficient electrical control of the magnetic state of ferromagnets, ferrimagnets and antiferromagnets. However, the conventional SOT has severe limitation that only in-plane spins accumulate near the surface, whether interpreted as a spin Hall effect (SHE) or as an Edelstein effect. Such a SOT is not suitable for controlling perpendicular magnetization, which would be more beneficial for realizing low-power-consumption memory devices. Here we report the observation of a giant magnetic-field-like SOT in a topological antiferromagnet Mn_3_Sn, whose direction and size can be tuned by changing the order parameter direction of the antiferromagnet. To understand the magnetic SHE (MSHE)- and the conventional SHE-induced SOTs on an equal footing, we formulate them as interface spin-electric-field responses and analyzed using a macroscopic symmetry analysis and a complementary microscopic quantum kinetic theory. In this framework, the large out-of-plane spin accumulation due to the MSHE has an inter-band origin and is likely to be caused by the large momentum-dependent spin splitting in Mn_3_Sn. Our work demonstrates the unique potential of antiferromagnetic Weyl semimetals in overcoming the limitations of conventional SOTs and in realizing low-power spintronics devices with new functionalities.

## Abstract (web summary)

Conventional spin-orbit torque (SOT) enables electrical control of in-plane spins, not suitable for perpendicular magnetization. Here, the authors observe a large magnetic-field-like SOT due to a large out-of-plane spin accumulation in topological antiferromagnet Mn_3_Sn.

## Introduction

In the conventional nonmagnetic materials with high crystalline symmetry used in SHE-related experiments, current-induced spin accumulation has its polarization parallel to the surface and perpendicular to the current direction^1–6^. Thus, the SOTs generated by the accumulated spins can only generate slow in-plane magnetization switching or precessional motion of out-of-plane magnetization^7^. Therefore, efficient switching in magnetic devices with perpendicular magnetic anisotropy can be achieved only by applying an undesirable bias magnetic field^8–10^ or an effective magnetic field^11–13^. It is nevertheless imperative to have directional control of the current-generated spin accumulation and its resulting SOTs to realize low-power spintronic devices utilizing magnetic thin films with perpendicular magnetic anisotropy. With this motivation, recent experimental and theoretical studies have demonstrated the generation of out-of-plane components in the spin accumulation by using the anomalous Hall effect (AHE) of the ferromagnet^14,15^, antiferromagnets^16,17^, ferromagnet/nonmagnet metal interface effects^18^, and low-symmetry materials such as transition-metal dichalcogenides (TMDs)^19,20^.

The antiferromagnetic Weyl semimetals (Weyl antiferromagnets) with chemical formula Mn_3_*X* (*X* = Sn, Ge) exhibit exotic physical properties such as the anomalous Hall effect^21^, the anomalous Nernst effect^22^, the magneto-optical Kerr effect^23^, and omnidirectional read-out^24^, despite having nearly compensated total spin magnetization. These properties are partly related to the large momentum-space Berry curvatures of their low-energy Weyl quasi-particles^25,26^. Remarkably, the magnetic states of these materials can be controlled by a small external magnetic field^21^ or a spin-orbit torque^27^, as if they were ferromagnets. It is thus anticipated that the topological Weyl antiferromagnet can offer new functionalities for spintronics. In fact, a novel magnetic spin Hall effect (MSHE) has been discovered in Mn_3_Sn, in which the spin-polarization direction of current-induced spin accumulation changes its sign upon flipping the chiral antiferromagnetic order of Mn_3_Sn^28^. Interestingly, the theoretical calculations hinted at a sizeable out-of-plane component of the current-induced spin density due to the MSHE i.e., out-of-plane MSHE. Very recently, in the non-collinear antiferromagnet Mn_3_GaN^16^ and the collinear antiferromagnet Mn_2_Au^17^, SOTs by out-of-plane spin component have been observed by means of temperature or strain-induced magnetic order control. However no experimental confirmation in the Weyl antiferromagnet Mn_3_*X* has been made to date.

Here we report the first experimental deomstration of out-of-plane MSHE and the associated giant field-like torque. Namely, its sign can be controlled by the direction of the staggered moments of the antiferromagnetic Weyl semimetal Mn_3_Sn. Conceptually, SOTs at the interface between two magnetic materials require a new classification scheme compared to the standard field-like torque (FLT) *vs*. anti-damping spin-transfer torque (STT) classification applied at a nonmagnetic-metal/ferromagnet interface, especially when the two magnetic order parameters can be separately manipulated^29^. Therefore, here we take a symmetry-based approach and construct a microscopic theory based on the interface spin to electric-field response approach adopted in ref. ^28^, without relying on spin currents and use it to separate STT and FLTs due to SHE and MSHEs.

## Results

### Detection of effective out-of-plane magnetic field due to MSHE

To investigate SOTs caused by the MSHE, the ST-FMR technique is applied to *single-crystal* Mn_3_Sn/Ni-Fe bilayers. ST-FMR is a powerful technique to evaluate current-induced SOTs in bilayer structures involving paramagnetic heavy metals^30^ or topological insulators^31,32^. A Mn_3_Sn rectangular strip with dimensions 10 × 0.24 × 55 μm^3^ was cut out of a single crystal using a focused ion-beam technique, followed by e-beam deposition of a 20-nm thick Ni-Fe layer on top of the strip surface.

**Fig. 1:**
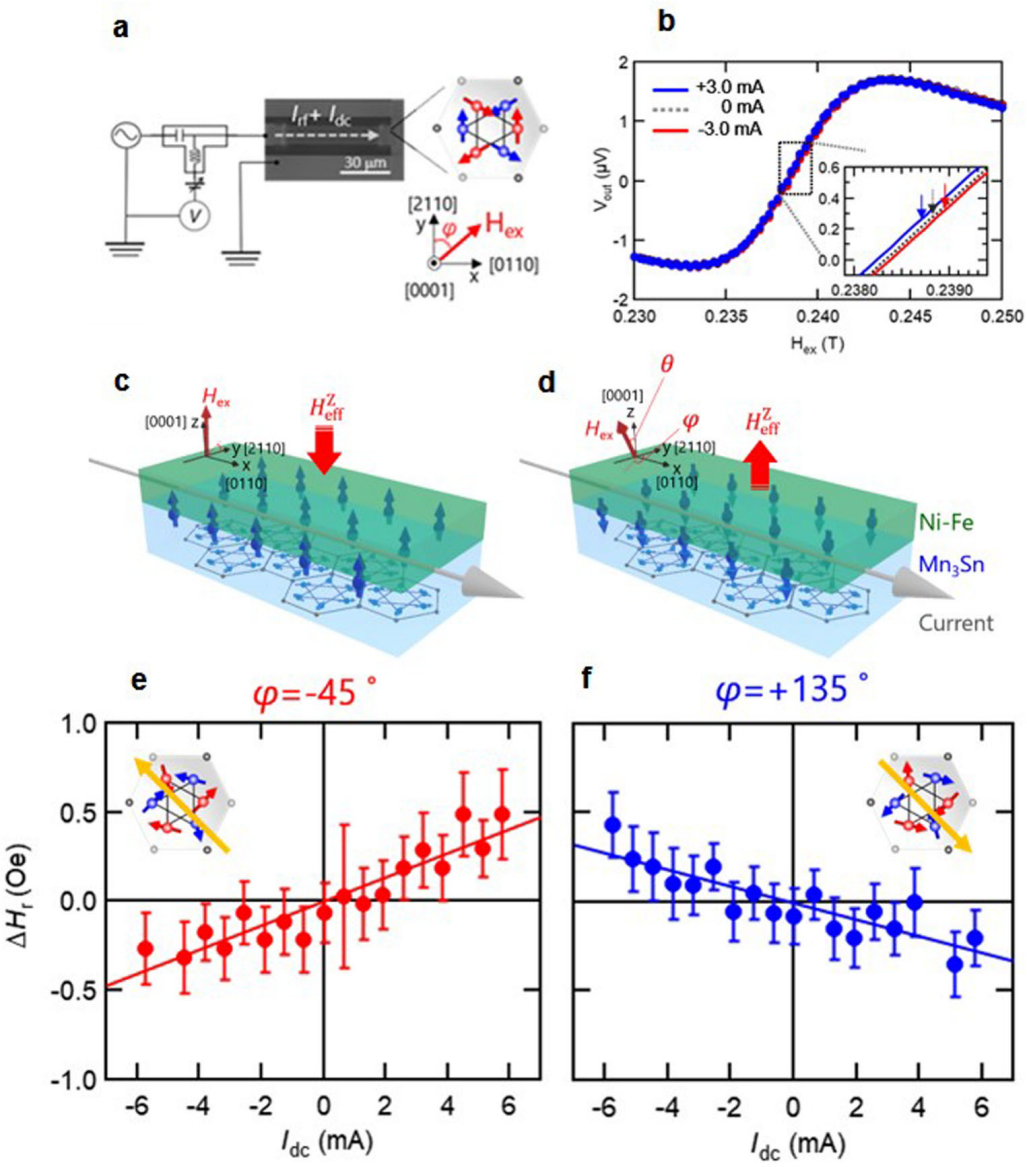
Detection of the effective field depending on the magnetic octupole orientation in Mn_3_Sn. **a** Measurement circuit, **b** ST-FMR resonance spectrum in Mn_3_Sn/Ni-Fe bilayer. **c**, **d** Schematic image of an out-of-plane component of spin accumulation at Mn_3_Sn/Ni-Fe interface. **e**, **f** Resonance field shift by dc current (**e** Δ*H*_r_=*H*_r_ (*φ*=−45°, *θ*=45°)-*H*_r_(*φ*=−45°, *θ* =135°), **f** Δ*H*_r_=*H*_r_ (*φ*=135°, *θ* =45°)-*H*_r_(*φ*=135°, *θ* =135°)) Error bars represent the standard deviation of five replicate measurements. Input radio frequency and power are 13 GHz and 7 mW.

Firstly, to verify the existence of an out-of-plane component of spin accumulation as predicted by theory^28^, we examine whether the spin accumulation can act as an effective out-of-plane magnetic field ${H}_{{{{{{\rm{eff}}}}}}}^{z}$ by measuring a shift of the resonance field *H*_r_ in the ST-FMR spectrum under the application of dc current. Fig. 1a and b respectively show the measurement circuit and a typical ST-FMR spectrum. We can see a small shift, which may include not only a contribution from ${H}_{{{{{{\rm{eff}}}}}}}^{z}$ but also that of the current induced in-plane magnetic field generated by the spin-accumulation due to the conventional SHE. We therefore excluded the influence of the current induced in-plane real and effective magnetic fields by focusing on the difference Δ*H*_r_ between shifts measured for out-of-plane magnetic fields at polar angles *θ* = 45° and 135° with fixed azimuthal angle φ. Fig. 1e shows Δ*H*_r_ as a function of *I*_dc_ at φ = −45°. The inset shows the spin structure in Mn_3_Sn for φ = −45°. A positive slope in the variation of Δ*H*_r_ with *I*_dc_ indicates that the ${H}_{{{{{{\rm{eff}}}}}}}^{z}$ is along the +z direction, as illustrated in Fig. 1c. On the other hand, the variation of Δ*H*_r_ with *I*_dc_ for φ = +135° clearly shows a sign change of the slope that we associate with the reversed spin structure of Mn_3_Sn. This observation demonstrates directional switching of the out-of-plane MSHE^28^.

### Angular dependence of in-plane torque τ^φ^ and the out-of-plane torque τ^z^

**Fig. 2:**
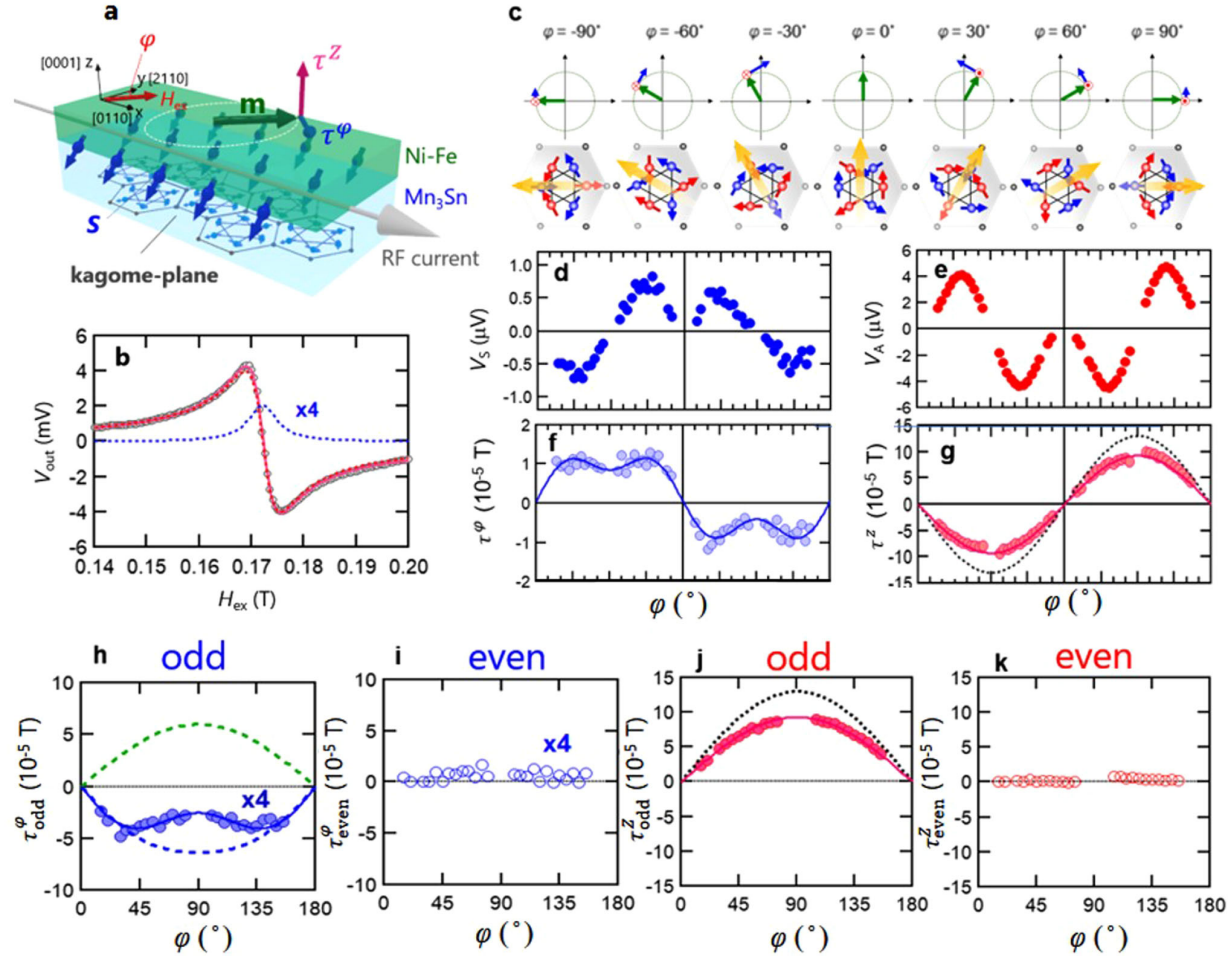
Spin-torque FMR measurements in a Mn_3_Sn/Ni-Fe bilayer. **a** Measurement setup for spin-torque FMR measurement (ST-FMR). The red and blue arrows on the respective top and bottom surface of the Mn_3_Sn stack indicates spin accumulation. **b** ST-FMR spectrum. **c** (Upper) Magnetization of Ni-Fe (green arrows) and detected in-plane torque τ^φ^ (blue arrows) and out-of-plane torque τ^Z^ (red) in Mn_3_Sn/Ni-Fe bilayer system; (Lower) Spin structure of Mn_3_Sn and cluster magnetic octupole (orange arrows). **d**, **e** In-plane magnetic field angle dependence of symmetric voltage *V*_S_ and Anti-symmetric voltages *V*_A_. **f**, **g** In-plane torque τ^φ^ and out-of-plane torque τ^Z^. **h**, **i** odd- and even-component of τ^φ^ under reversal of the ferromagnetic magnetization **m**. Green (Black) dashed line corresponds to τ^φ^ due to conventional SHE (MSHE). **j**, **k** Odd- and even-component of τ^z^. Black dashed line corresponds to τ^z^ due to Oersted field.

Notably, the above results demonstrate that the MSHE in Mn_3_Sn exerts a SOT on the adjacent ferromagnetic layer that can be tuned by changing the orientation of the magnetic octupoles. Therefore we have next studied the direction and magnitude of the SOT that depends on the magnetic octuopole orientation in Mn_3_Sn by measuring the in-plane azimuthal angle φ dependence of ST-FMR spectrum in a Mn_3_Sn/Ni-Fe bilayer. Fig. 2a illustrates the measurement circuit together with a schematic explanation of the in-plane torque τ^φ^ and the out-of-plane torque τ^z^ exerted on the Ni-Fe magnetization in the Mn_3_Sn/Ni-Fe bilayer. When an rf current is applied to the bilayer stripe, the induced rf Oersted field ${H}_{{{{{{\rm{Oe}}}}}}}^{{{{{{\rm{rf}}}}}}}\,$excites FMR in the top Ni-Fe layer under the application of external field *H*_ex_. The spin accumulation simultaneously appears at the Mn_3_Sn/Ni-Fe interface, which exerts SOTs linear in the rf current on the Ni-Fe magnetization.

Fig. 2b shows a typical ST-FMR spectrum of a Mn_3_Sn/Ni-Fe bilayer. All measurements were carried out at room temperature. The input radio frequency and power are 13 GHz and 7 mW, respectively. The ST-FMR spectrum generally contains a symmetric component ${V}_{{{{{{\rm{S}}}}}}}=-\frac{1}{4}\frac{{dR}}{d\varphi }\frac{\gamma {I}_{{{{{{\rm{rf}}}}}}}{\sin}\;\varphi }{\delta 2\pi {\left({df}/{dH}\right)}_{{H}_{{{{{{\rm{ex}}}}}}}={H}_{{{{{{\rm{r}}}}}}}}}S\frac{\delta }{{\delta }^{2}+{({H}_{{{{{{\rm{ex}}}}}}}-{H}_{{{{{{\rm{r}}}}}}})}^{2}}$ and an asymmetric component, ${V}_{{{{{{\rm{A}}}}}}}=-\frac{1}{4}\frac{{dR}}{d\varphi }\frac{\gamma {I}_{{{{{{\rm{rf}}}}}}}{\sin }\;\varphi }{\delta 2\pi {\left({df}/{dH}\right)}_{{H}_{{{{{{\rm{ex}}}}}}}={H}_{{{{{{\rm{r}}}}}}}}}A\frac{({H}_{{{{{{\rm{ex}}}}}}}-{H}_{{{{{{\rm{r}}}}}}})}{{\delta }^{2}+{({H}_{{{{{{\rm{ex}}}}}}}-{H}_{{{{{{\rm{r}}}}}}})}^{2}}\,$. Here dR/dφ, *γ*, *I*_rf_, φ, δ, *S* and *A* are respectively the sample resistance change due to precessional motion, the gyromagnetic ratio, the input rf current, the in-plane magnetic field angle, the half-width at half maximum of the ST-FMR spectrum and the coefficients of the symmetric and asymmetric components^30,33,34^. The torques (τ^φ^ and τ^z^) per moment exerted on the Ni-Fe layer measured in Tesla can be obtained from the values of *V*_S_ and *V*_A_ through ${\tau }^{\varphi (z)}=-8\pi {V}_{S(A)}\delta (\frac{df}{dH})/\{(\frac{dR}{d\varphi })\gamma {I}_{rf}\}$. Note that *V*_S_ and *V*_A_ respectively correspond to τ^φ^ and τ^z^ regardless of the latter’s microscopic mechanism. In the case of SOTs due to a *conventional* SHE in the diffusive transport regime, the bulk spin current in the nonmagnetic metal leads to interface spin accumulation polarized along the *y*-axis even in the absence of the ferromagnetic layer, which gives rise to ${\tau }^{z}\propto \left({{{{{\bf{m}}}}}}{{{{{\boldsymbol{\times }}}}}}\delta {s}_{y}{\hat{{{{{{\bf{e}}}}}}}}_{y}\right)\cdot{{{{{\boldsymbol{\bullet }}}}}}{\hat{{{{{{\bf{e}}}}}}}}_{z}$, where **m** and δ**s** are the magnetization directions of the ferromagnet and the spin accumulation, respectively. The torque τ^z^, in this case, is a purely FLT since it is odd under **m→-m**. The spin current from both the bulk of the nonmagnetic layer and the diffusion of the accumulated spins into the ferromagnetic layer then leads to the STT ${{{{{{\boldsymbol{\tau }}}}}}}^{\varphi }\propto {{{{{\boldsymbol{\propto }}}}}}{{{{{\bf{m}}}}}}{{{{{\boldsymbol{\times }}}}}}(\delta {s}_{y}{\hat{{{{{{\bf{e}}}}}}}}_{y}{{{{{\boldsymbol{\times }}}}}}{{{{{\bf{m}}}}}})$. The torque **τ**^φ^ is therefore purely a STT since it is even under the reversal of **m**. In our discussion below, the scalar τ^φ^ is the in-plane torque projected to the direction of ${\hat{{{{{{\bf{e}}}}}}}}_{z}{{{{{\boldsymbol{\times }}}}}}{{{{{\bf{m}}}}}}$ and therefore has opposite parity to **τ**^φ^ under **m** reversal. However, in more general cases with lower symmetry, τ^z^ and τ^φ^ are neither purely even nor odd under **m→−m**. For example, a $\delta {s}_{z}$ spin accumulation can lead to a field-like **τ**^φ^. A further complication in the present system comes from the nontrivial dependence of the accumulated spin on the order parameter direction of Mn_3_Sn.

To separate the different contributions to τ^z^ and τ^φ^ in the present system, we have measured their dependence on the in-plane magnetic field direction i.e., the azimuthal angle φ. The plane of the external magnetic field *H*_ex_ rotation corresponds to the *kagome* plane in Mn_3_Sn. Fig. 2c shows the schematic illustration of spin structures together with the octupole orientation in Mn_3_Sn, aligned along an external magnetic field that is larger than the switching field of Mn_3_Sn^28^. Fig. 2d and e show the azimuthal angle φ dependence of the magnitudes of *V*_S_ and *V*_A_ in the Mn_3_Sn/Ni-Fe bilayer, from which one can obtain τ^φ^ and τ^z^ as shown in Fig. 2f and g.

The angular dependence of τ^φ^ in the Mn_3_Sn/Ni-Fe bilayer (Fig. 2f) is entirely different from that of STT due to *conventional* SHE (Supplementary Fig. 1). The *conventional* SHE induced STT signal exhibits a simple sin φ dependence due to the projection of ${{{{{{\boldsymbol{\tau }}}}}}}^{\varphi }\propto {{{{{\boldsymbol{\propto }}}}}}{{{{{\bf{m}}}}}}{{{{{\boldsymbol{\times }}}}}}(\delta {s}_{y}{\hat{{{{{{\bf{e}}}}}}}}_{y}{{{{{\boldsymbol{\times }}}}}}{{{{{\bf{m}}}}}})$ onto ${\hat{{{{{{\bf{e}}}}}}}}_{z}{{{{{\boldsymbol{\times }}}}}}{{{{{\bf{m}}}}}}$. Such a τ^φ^ reaches a maximum when the magnetization of Ni-Fe is (anti) parallel to the rf charge current, i.e. φ = ±90° (Supplementary Fig. 1). However, the τ^φ^ in Mn_3_Sn/Ni-Fe bilayer has local maxima and minima at around φ = ±45° and ±135°. The angular variations of the direction and amplitude of the experimentally determined **τ**^φ^ are illustrated in the upper row of Fig. 2c.

### Analysis based on interface spin-electric-field responses

The unusual angular dependence in the present system calls for a revisit of the conventional spin-current picture of SOT in bilayer structures. In ref. ^28^, we have defined the (M)SHE as the boundary spin to electric-field response, avoiding conceptual difficulties that arise in defining both spin currents in strongly spin-orbit-coupled systems and spin-currents that flow in the direction perpendicular to a 2D system. In the case of the SOT, one can still define τ as the torque due to the current- or electric-field-induced spin density δ**s**^28^, since it is exchange-coupled to the magnetization **m** of the ferromagnetic layer. In this description, the FLT and STT in *conventional* ferromagnetic/nonmagnetic-metal bilayers correspond respectively to δ**s** contributions that are even and odd under **m→**-**m**^35–37^. When the nonmagnetic metal is replaced by a magnetic one, such as Mn_3_Sn, δ**s** additionally depends on its magnetic order parameter denoted as **n**. The SHE and the MSHE can be distinguished by their parity under **n→**-**n**. We can therefore describe STTs and FLTs due to the SHE and the MSHE by using a single spin-electric-field response function, and examining its dependence on the *separate* reversal of **m** and **n** (Table S1). For example, the STT (FLT) due to the MSHE results is the part of δ**s** that is odd (even) in **m** and *odd* in **n**.

Based on this new picture, we performed symmetry analysis of the spin-electric-field response function χ, defined through δ**s** = χ **E** with **E** being the electric field, at the Ni-Fe/Mn_3_Sn interface, which has a *C*_3*v*_ symmetry determined by Mn_3_Sn since the Ni-Fe layer is polycrystalline. By truncating the Fourier expansion in order parameter direction at 2^nd^ order, and taking into account the opposite rotation of the weak magnetization to the sublattice moments of Mn_3_Sn, we obtain the following angular dependence of $\delta {s}_{z}$ corresponding to the FLT component of the MSHE:

1 $$\delta {s}_{z}=\left({b}_{2}+{d}_{5}{{{\sin }}}^{2}\varphi +{d}_{6}{{{\cos }}}^{2}\varphi \right){{\sin }}\varphi$$

where b_2_, d_5_, and d_6_ are system-dependent parameters. ${\tau }_{{{{{{\rm{odd}}}}}}}^{\varphi }$ has the same angular dependence since ${\tau }_{{{{{{\rm{odd}}}}}}}^{\varphi }\propto \left({{{{{\boldsymbol{m}}}}}}\times \delta {s}_{z}{\hat{{{{{{\bf{e}}}}}}}}_{z}\right)\cdot{{{{{\boldsymbol{\cdot }}}}}}\left({{{{{\boldsymbol{m}}}}}}\times {\hat{{{{{{\bf{e}}}}}}}}_{z}\right){{{{{\boldsymbol{\propto }}}}}}\propto \delta {s}_{z}$. However, one should note that the $\delta {s}_{z}$ corresponding to the STT component of the SHE also has the same form of angular dependence as in Eq. (1), and cannot be separated from the latter in this symmetry approach. On the other hand, the $\delta {s}_{z}$ contributing to ${\tau }_{{{{{{\rm{even}}}}}}}^{\varphi }$ has the angular dependence of ${c}_{4}{{\cos }}2\varphi +{c}_{8}$, where the two terms are respectively due to the FLT of SHE and the STT of MSHE, provided that the truncation of the order parameter directions at 2nd order is approximately valid.

To complement the symmetry analysis with a microscopic formalism for calculating the STT and FLT of (M)SHE separately, we adopt the quantum kinetic theory with the constant relaxation time (*τ*) approximation as in ref. ^28^. In this approximation the O(τ^0^) contribution to χ includes the STT component of the SHE and the FLT component of the MSHE; whereas the O(τ^1^) contribution includes the FLT component of the SHE and the STT of the MSHE (Table S2). The SHE and MSHE contributions in each of these can be further separated by, e.g., treating the exchange field term in the ferromagnetic layer as a perturbation, controlled by a dimensionless perturbation parameter λ_F_. Then the terms with even (odd) powers of λ_F_ in χ are even (odd) under reversal of the ferromagnetic magnetization **m** alone. In practice, since properties of the ferromagnetic layer itself in ST-FMR experiments usually have a very simple dependence on its magnetization direction, we argue that it is likely to be sufficient to stop at the first order in λ_F_. The expressions for the four contributions to χ obtained by perturbing the equilibrium Liouvillian of the bilayer system under the above approximations are given in the SI, which we apply to a simple model below.

Fig. 2h and i show the odd (${\tau }_{{{{{{\rm{odd}}}}}}}^{\varphi }$) and even (${\tau }_{{{{{{\rm{even}}}}}}}^{\varphi }$) components of τ^φ^ from the experimental data. The odd component under **m** reversal is well fit by Eq. (1) from the symmetry analysis, with b_2_, d_5_, and d_6_ equal to −2.0, 1.3, and −0.7 (mT/A), respectively. However, such numbers are still much smaller than the τ^φ^ estimated from the experimental results of *H*_r_ shift in Fig. 1, which is about −83.3 ± 12.1 (mT/A). We show below that this discrepancy is caused by the suppression of τ^φ^ by partial cancellation between the STT of the SHE and the FLT of the MSHE. Moreover, ${\tau }_{{{{{{\rm{even}}}}}}}^{\varphi }$ is negligibly small compared to ${\tau }_{{{{{{\rm{odd}}}}}}}^{\varphi }$, whereas the difference between the | $\Delta {H}_{{{{{{\rm{r}}}}}}}/{I}_{{{{{{\rm{dc}}}}}}}\,$| in Fig. 1e and f (of about 10%) which is produced by the FLT of the SHE is considerable. This implies that the $\delta {s}_{z}$ due to the STT of the MSHE and the FLT of the SHE must partially cancel, as discussed further below.

A similar decomposition into odd (${\tau }_{{{{{{\rm{odd}}}}}}}^{z}$) and even (${\tau }_{{{{{{\rm{even}}}}}}}^{z}$) components of the out-of-plane torque τ^z^ from the experimental data is shown in Fig. 2j and k. Like τ^φ^, the even component of τ^z^ is vanishingly small, indicating that the FLT of the MSHE and the STT of the SHE are both small in this Mn_3_Sn/Ni-Fe bilayer, or that they partially cancel. In contrast, the ${\tau }_{{{{{{\rm{odd}}}}}}}^{z}$ (red circles in Fig. 2j), although much larger, has a dominant contribution from the rf current-induced Oersted field ${\tau }_{{{{{{\rm{Oe}}}}}}}^{z}$. The latter is indicated by the black dotted curves in Fig. 2j and was estimated from measurements in a control sample. Aside from ${\tau }_{{{{{{\rm{Oe}}}}}}}^{z}$, ${\tau }_{{{{{{\rm{odd}}}}}}}^{z}$ has contributions from the STT of MSHE and the FLT of SHE. However, from the *H*_r_ shift measurement by dc current at *θ* = 90°, we found that the contribution of FLT of SHE is very small (Supplementary Fig. 3). Thus, the dominant contribution to ${\tau }_{{{{{{\rm{odd}}}}}}}^{z}-{\tau }_{{{{{{\rm{Oe}}}}}}}^{z}$ is from the $\delta {s}_{x,y}$ due to the STT of MSHE.

### Modulation of magnetic damping constant by τ_STT_

**Fig. 3:**
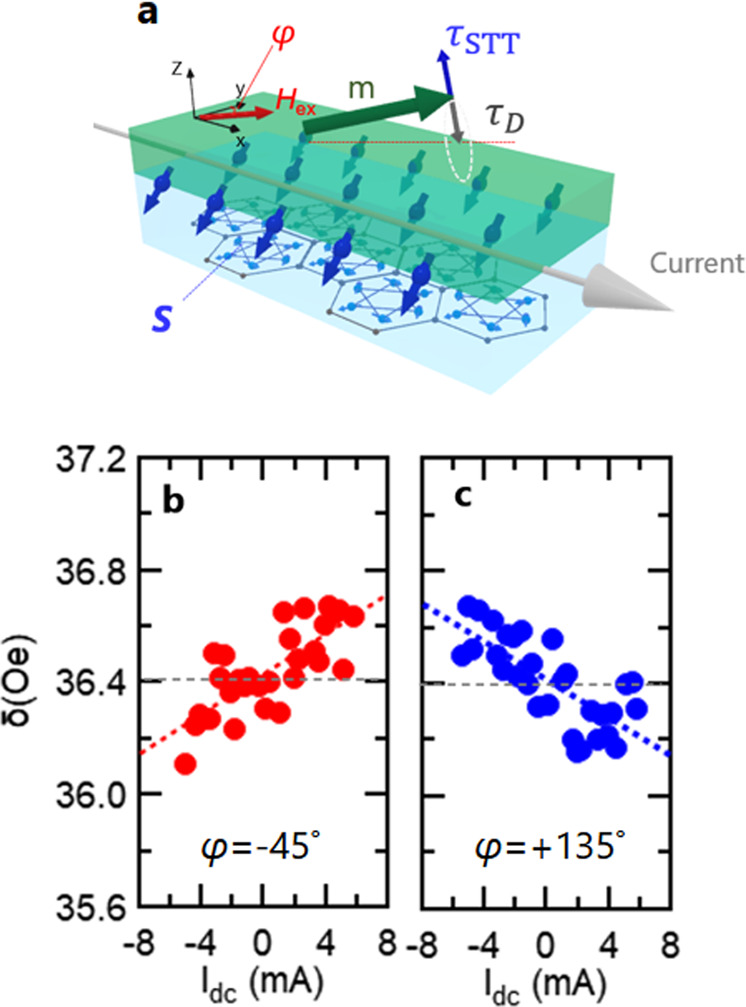
Modulation of effective magnetic damping by spin Hall effect in Mn_3_Sn. **a** Schematic image of modulation of magnetic damping torque τ^D^ by STT τ_STT_ due to conventional SHE in Mn_3_Sn. The polar angle *θ* is fixed at 90°. **b**, **c** FMR line width δ at φ= −45°(**b**) and +135° (**c**) as a function of applied dc current.

To separate the more conventional SHE-induced STT from ${\tau }_{{{{{{\rm{odd}}}}}}}^{\varphi }$, we measured the modulation of magnetic damping constant by dc current. Fig. 3a shows a schematic image of the modulation of magnetic damping torque τ_D_ by τ_STT_. We set the polar angle *θ* to 90°so that τ_D_ is modulated by ${\tau }_{{{{{{\rm{odd}}}}}}}^{\varphi }$ of the STT due to the SHE. Fig. 3b, c show experimental results of FMR linewidth δ at φ = −45° and +135° as a function of applied dc current. We observed linear dependence of δ on the dc current, with its slope opposite between φ = −45° and +135°, consistent with the behavior of the SHE-induced STT. From these data we estimate the spin torque τ_STT_ through the formula ${\tau }_{{{{{{\rm{STT}}}}}}}=\frac{\gamma \varDelta \delta ({H}_{{ext}}+{M}_{{eff}}/2)}{2\pi {f\;cos}\;\varphi \; {Ic}({Mn}3{Sn})}$, here ∆δ is the dc current-induced change of FMR linewidth. The obtained τ_STT_ is 75.0 ± 6.1(mT/A), which is comparable with the in-plane FLT due to MSHE estimated from the *H*_r_ shift in Fig. 1. These experimental results strongly support our hypothesis that the τ^φ^ contributed by the STT component of the SHE suppresses the FLT of MSHE in the same direction in the present system.

### Suppression of FLT contribution by inserting the thin Cu layer

**Fig. 4:**
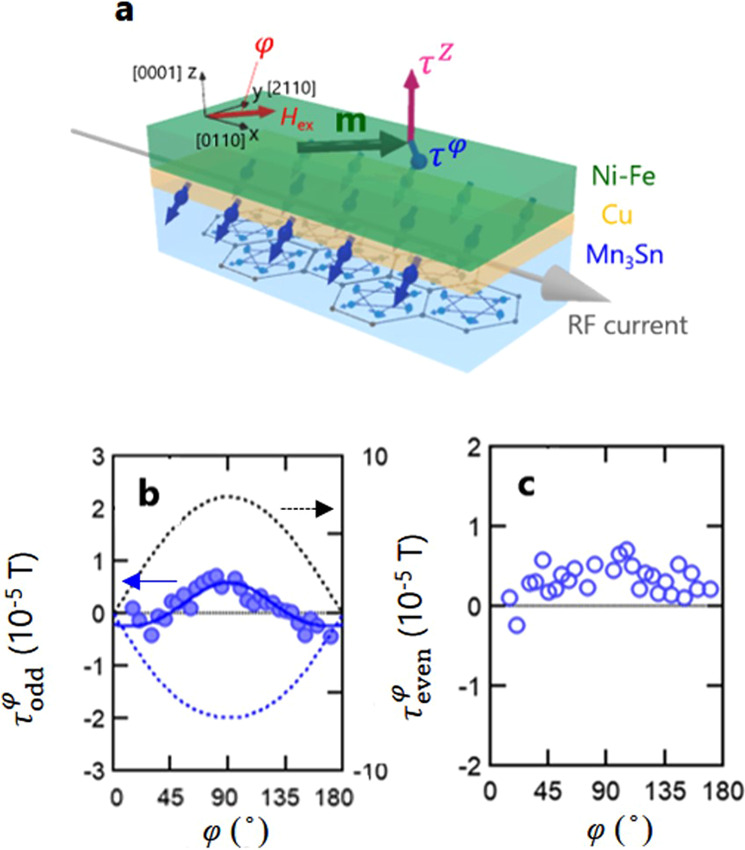
Spin-torque FMR measurements in Mn_3_Sn/Cu/Ni-Fe trilayer. **a** Measurement setup for ST-FMR in Mn_3_Sn/Cu/Ni-Fe trilayer. The red and blue arrows on the respective top and bottom surfaces of the Mn_3_Sn stack indicate spin accumulation. The red and blue arrows on the Ni-Fe magnetization **m** correspond to out-of-plane torque τ^z^ and in-plane torque τ^φ^. **b**, **c** odd-(**b**) and even-(**c**) components of τ^φ^ as a function of the in-plane **m** direction φ. Blue (Black) dashed line corresponds to τ^φ^ due to the STT of the *conventional* SHE (FLT of the MSHE).

To provide further evidence supporting the hypothesis above, we measured the SOT in a Mn_3_Sn(140)/Cu(5)/Ni-Fe(15) trilayer sample (Fig. 4a). If τ^φ^ due to the FLT of MSHE nearly compensates that due to the STT of SHE as shown in Fig. 2h, inserting the thin Cu layer between Mn_3_Sn and Ni-Fe layers should change the angular dependence of the observed τ^φ^ because the FLT is expected to be more strongly suppressed than the STT by a Cu spacer^38^. Indeed, Fig. 4b shows that ${\tau }_{{{{{{\rm{odd}}}}}}}^{\varphi }$ in the presence of the spacer has a very different angular dependence from that in Fig. 2h, and even has a sign change at φ=90°. Moreover, ${\tau }_{{{{{{\rm{even}}}}}}}^{\varphi }$ is even enhanced after Cu insertion compared to Fig. 2i. Both of these changes support our argument that there exists strong compensation between FLTs and STTs along the same directions in Mn_3_Sn/Ni-Fe bilayer. This scenario can also be captured by using a toy model mimicking the Mn_3_Sn/Ni-Fe interface and applying the quantum kinetic theory results for χ. Qualitatively, one expects ${\tau }_{{{{{{\rm{odd}}}}}}}^{\varphi }$ due to either SHE or MSHE to have a dominant sin⁡φ dependence according to Eq. (1), when spin-orbit coupling can be viewed as a perturbation. The unusually small sin⁡φ component compared to higher-order angular variations suggests accidental cancellation of the leading-order terms due to SHE and MSHE separately.

### Comparison spin torque ratio with previous reports

**Fig. 5:**
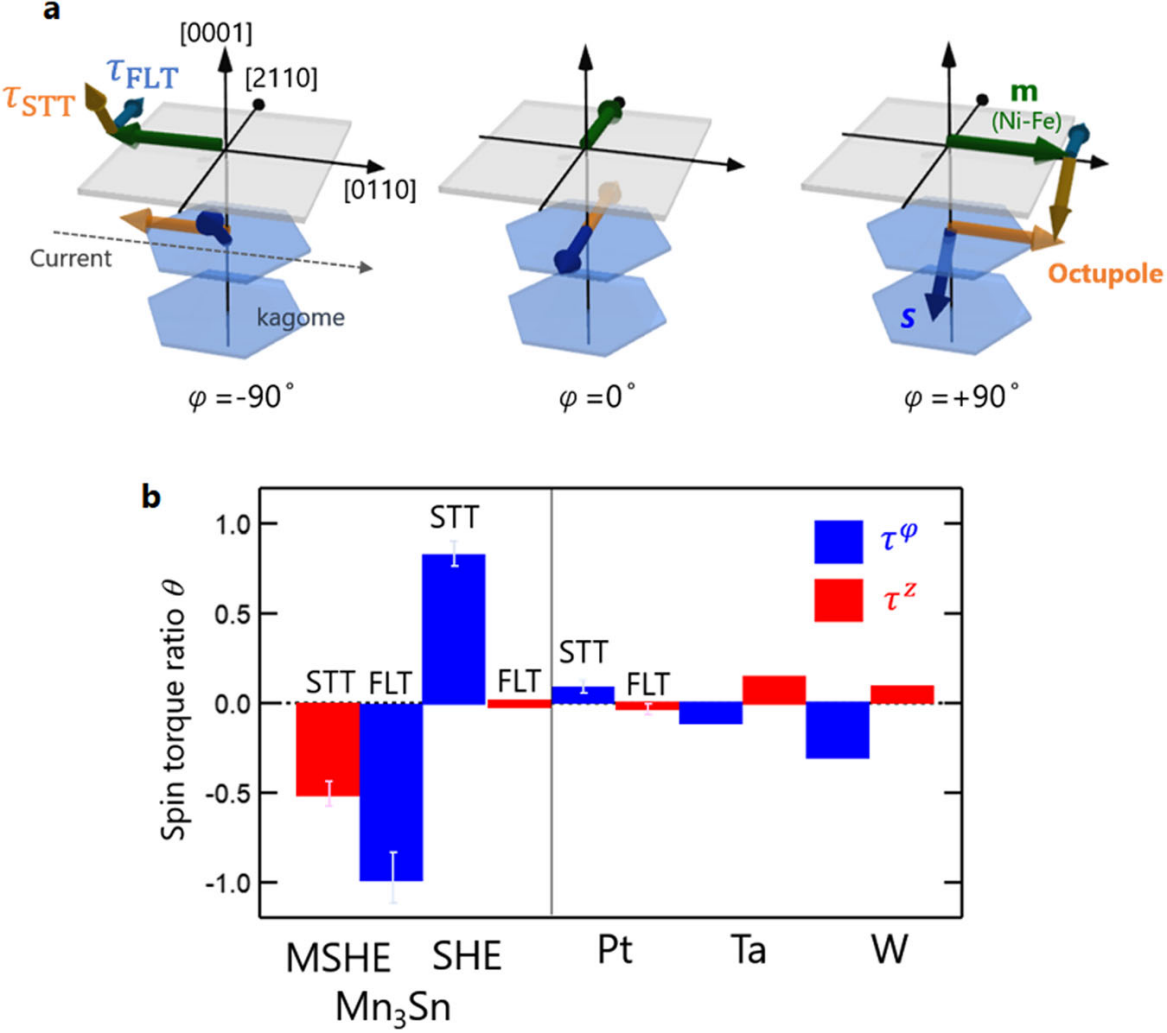
Giant field-like torque and spin polarization vector of spin accumulation in Mn_3_Sn/Ni-Fe bilayer. **a** Schematic images of spin polarization vector **S** (blue arrow) of spin accumulation at Mn_3_Sn/Ni-Fe interface and spin transfer torque (STT) and field-like torque (FLT) as a function of the in-plane direction φ of the magnetization **m** (green arrow) in Ni-Fe layer. **b** Comparisons of spin torque ratio θ in Mn_3_Sn and that in typical transition metals. Blue and red bars correspond to τ^φ^ and τ^z^ at φ = 90°. The sign of spin torque due to MSHE is changed for φ = −90°.

Fig. 5a shows schematic illustrations of the angular dependence of τ_STT_ and τ_FLT_ in a Mn_3_Sn/Ni-Fe bilayer, obtained by taking all the experimental data and analyses above into account. The out-of-plane (in-plane) component of τ_STT_ is mainly due to the MSHE (SHE), while the τ_FLT_ has a dominant in-plane component mostly contributed by the MSHE. For a qualitative comparison with the SOT in conventional bilayer systems, we also plotted in Fig. 5a spin accumulation **S** under the assumption that τ_STT_ can be understood as due to the diffusion of **S** into the FM layer. Such a representation shows that the polarization direction of the spin accumulation at the Mn_3_Sn surface is tilted out of the *xy* plane.

Finally, a quantitative comparison is made between the spin torque ratio θ in the present system and those in previous reports (Fig. 5b) by means of the same manner with SHE^30^. ${\theta }_{{{{{{\rm{FLT}}}}}}({{{{{\rm{STT}}}}}})}=\frac{\left(\frac{2e}{\hslash }\right){\sigma }_{{{{{{\rm{S}}}}}}}^{{{{{{\rm{FLT}}}}}}\left({{{{{\rm{STT}}}}}}\right)}}{{\sigma }_{{{{{{\rm{C}}}}}}}}=\frac{\left(\frac{2e}{\hslash }\right){\tau }_{{{{{{\rm{FLT}}}}}}({{{{{\rm{STT}}}}}})}{M}_{{{{{{\rm{S}}}}}}}{t}_{{{{{{\rm{FM}}}}}}}}{S}$, where ${\sigma }_{{{{{{\rm{C}}}}}}({{{{{\rm{S}}}}}})}$ and *S* are the charge conductivity, spin current conductivity in Mn_3_Sn and cross-sectional area onf Mn_3_Sn layer. The θ, in particular the FLT due to the MSHE, are more substantial than the reported values in paramagnetic metals such as Pt^30,39,40^, *β*-Ta^8,41^, *β*-W^9,42^. Estimated spin current conductivity ${\sigma }_{{{{{{\rm{S}}}}}}}^{{{{{{\rm{FLT}}}}}}}$ due to MSHE is 2.8 ± 0.4 × 10^5^ (*ħ*/2e Ω^−1^m^−1^), which is comparable with the calculated values in chiral antiferromagnets^40,43,44^. We note much larger FLT comes from the MSHE than from the SHE in Mn_3_Sn. This is because the former has an inter-band nature and could benefit from the strong momentum-dependent spin splitting in Mn_3_Sn. Giant FLTs due to the SHE have been observed in topological insulator/FM^45,46^ and TMD material/FM heterostructures^20,47,48^, which usually involve quasi-2D systems with large spin-orbit coupling at the interfaces. In near future, by utilizing the high-quality Mn_3_*X* thin film/ferromagnetic heterostructure, the giant FLT, as well as the STT due to bulk Mn_3_Sn, should directly contribute to realizing field-free magnetization switching^49^, high-speed magnetization control^7^, and domain wall manipulation^50^. Our findings thus provide a route for efficient manipulation of magnetic states and realization of novel functionalities by utilizing topological Weyl antiferromagnets.

## Methods

### Sample fabrication

The single crystal Mn_3_Sn used in the present study was prepared by using the Bridgman technique. The rectangular element, cut out of the single crystal using the Focused Ion Beam, transferred to the co-planar waveguide of 5-nm Ti /200-nm Au on a thermally oxidized Si substrate by using a W needle manipulator. The surface of the Mn_3_Sn element was cleaned by Ar-ion milling to remove the residual damaged layer. The NiFe and Al_2_O_3_ layer was formed by optical photolithography and lift-off process. The resistivity of the processed Mn_3_Sn crystal was 360 μΩ cm at room temperature, which is comparable to the bulk sample^14^.

### ST-FMR measurement set up

An rf current is applied along the long edge of the rectangle by a microwave analog signal generator (Keysight: MXG N5183A). An external static magnetic field *H*_ext_ in the range from 0 to 4.0 kOe is also applied. All the experiments are performed at room temperature. For resonance field shift measurement in Fig. 1, we used the dc current source (Yokogawa GS200) and lock in amplifier (NF LI5640).

## Supplementary information

Supplementary Information

## Data Availability

The datasets generated and analyzed during this study are available from the corresponding authors on reasonable request.

## Code availability

The corresponding computer codes are available from the corresponding authors on reasonable request.
