# Supplementary material for: Giant field-like torque by the out-of-plane magnetic spin Hall effect in a topological antiferromagnet
Source: Nat Commun. 2021 Nov 18;12:6491. doi: 10.1038/s41467-021-26453-y (PMC8602386; doi:10.1038/s41467-021-26453-y)
Supplement: Supplementary file 1 — Supplementary Information [file 41467_2021_26453_MOESM1_ESM.pdf]

Supplemental Information:

Giant field-like torque by the out-of-plane magnetic spin Hall effect in a topological antiferromagnet

Kouta Kondou[1,2*], Hua Chen[3,4], Takahiro Tomita[2,5], Muhammad Ikhlas[2,5],Tomoya Higo[2,6], Allan H. MacDonald[7], Satoru Nakatsuji[2,5,6,8,9], and YoshiChika Otani[1,2,5,8*]

[1]*RIKEN, Center for Emergent Matter Science (CEMS), Saitama 351-0198, Japan*

[2]*CREST, Japan Science and Technology Agency (JST), Kawaguchi, Saitama 332-0012, Japan*

[3]*Department of Physics, Colorado State University, Fort Collins, CO, USA.*

[4]*School of Advanced Materials Discovery, Colorado State University, Fort Collins, CO, USA*

[5]*Institute for Solid State Physics, The University of Tokyo, Kashiwa, Chiba 277-8581, Japan*

[6]*Department of Physics, University of Tokyo, Hongo, Bunkyo-ku, Tokyo 113-0033, Japan*

[7] *Department of Physics, University of Texas at Austin, Austin, TX, USA.*

[8]*Trans-scale Quantum Science Institute, University of Tokyo, Tokyo, Japan*

[9]*Institute for Quantum Matter and Department of Physics and Astronomy, Johns Hopkins University, Baltimore, Maryland 21218, USA*

*** Corresponding author: kkondou@riken.jp, yotani@issp.u-tokyo.ac.jp**

1. **Angular dependence of SOTs in conventional SHE of Pt**
2. **Resonace field shift as function of appied out-of-plane magnetic field**
3. **Contribution of FLT due to SHE in Mn3Sn/NiFe bilayer**
4. **Symmetry analysis of the spin-electric-field response in the presence of two magnetic order parameters**
5. **Quantum kinetic theory of the spin-transfer torque in terms of the spin-electric-field response function**
6. **Toy model calculation of the STT and FLT in NiFe/Mn_3Sn bilayer**

Note 1 Angular dependence of SOTs in conventional SHE of Pt

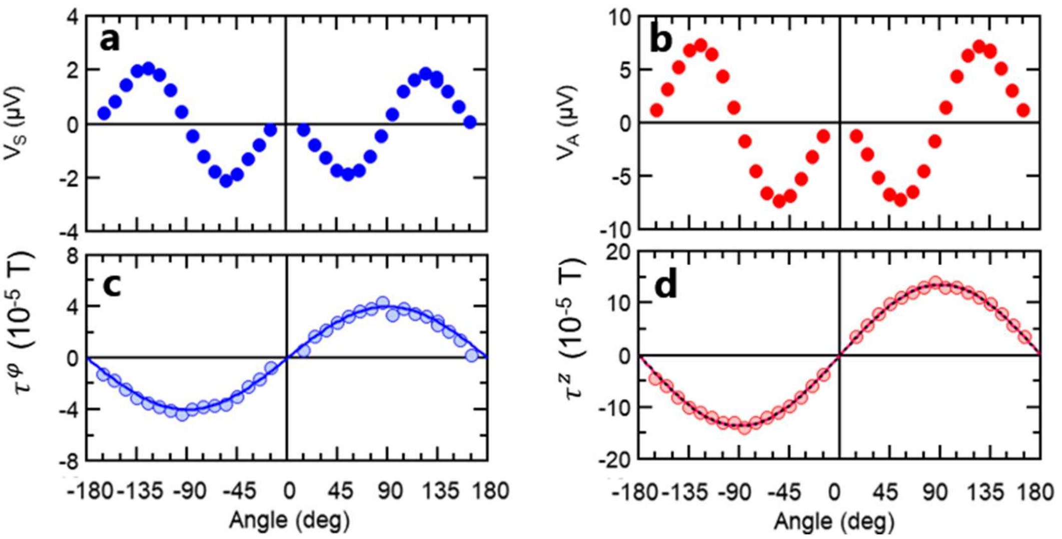

Supplementary Fig. 1| Angular dependence of the symmetric voltage V_S and asymmetric voltage V_A in Pt/Ni-Fe bilayer

A typical ST-FMR spectrum for Pt(10-nm)/Ni-Fe(10-nm) bilayeris shown. The input radio frequency and power are 13 GHz and 7 mW, respectively. Since the accumulated spins due to the conventional SHE in Pt are always aligned along the *y*-axis, the τ^{φ} and τ^{z} are respectively caused by the spin transfer torque τ_{STT} ($\mathbf{m}\times(S_y\times\mathbf{m})$) and the field-like torque τ_{FLT} ($\mathbf{m}\times S_y$). In the case of the conventional SHE of Pt, τ^{φ} varies as $\sin\theta$ as shown in Supplementary Fig. 1**c**, since the direction of spin polarization is always fixed along the *y*-axis [1]. In other words, when the magnetization of Ni-Fe is (anti) parallel to the charge current , i.e. $\varphi = \pm 90^{\circ}$, τ^{φ} takes its maximum value. Supplementary Fig. 1**d** shows the τ^{z} estimated from V_A. τ^{z} is comparable with the AC Oersted field plotted as a black dashed line. It follows that τ_{FLT} is negligibly small in this Pt/Ni-Fe bilayer.

Note 2 Resonance field shift versus appied out-of-plane magnetic field

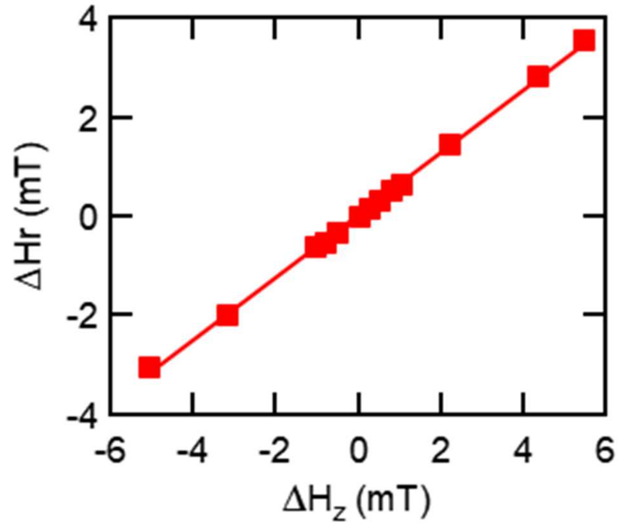

Supplementary Fig. 2 | Shift of the resonace field of the ST-FMR spectrum as a function of applied out-of-plane magnetic field

ST-FMR resonance field shift *vs.* out-of-plane magnetic field. Input radio frequency and power are 13 GHz and 7 mW. In-plane magnetic field angle φ is fixed at -45˚. $\Delta H_r = 0$ corresponds to θ=+45 ˚. From this measurement, we found that ΔHr is propotional to ΔH_z. In Fig.1 of the main text, we observed the ΔH_r of about 0.03-0.05 mT by dc charge current. The amplitude of ΔH_r thus corresponds to ΔH_z of 0.048-0.079 mT.

.

Note 3 Contribution of FLT due to the SHE in Mn_3Sn/NiFe bilayer

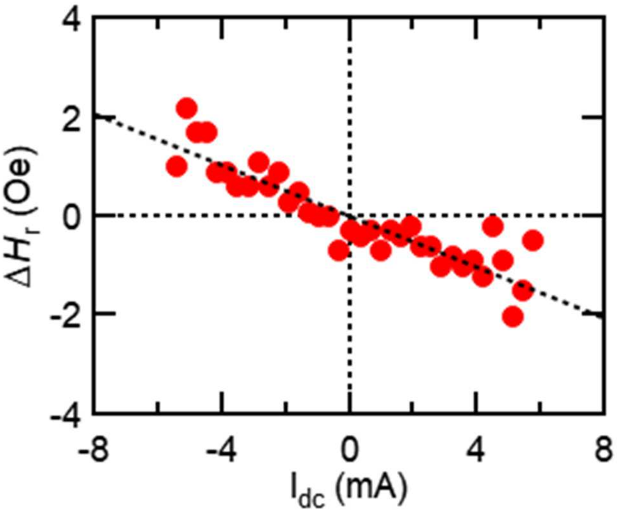

Supplementary Fig. 3| Modulation of the resonance field by dc charge current

Magnetic field angle is set to φ = -45˚ and θ=90˚. The black dashed line plots the resonance field shift by the Oersted field due to the dc charge current. The experimental plots follow the black broken line, which implies that the contribution of the FLT due to the SHE is smaller than error bar.

Note 4 Symmetry analysis of the spin-electric-field response in the presence of two magnetic order parameters

As argued in [2], it is conceptually more straightforward to formulate the SHE in terms of the spin density response to electric fields at the sample boundary, than to use the spin current language. At ferromagnet (FM)/nonmagnetic-metal (NM) interfaces the same picture has already been routinely used in first-principles theories of current-induced torques [3, 4]. The spin torque acting on the magnetization $\mathbf{M}$ is defined as $\boldsymbol{\tau} = \mathbf{M} \times \mathbf{B}_{\text{eff}} = \mathbf{M} \times (J\delta\mathbf{s})$, where $\delta\mathbf{s}$ is the current-induced spin density and J denotes the exchange coupling between $\mathbf{M}$ and $\mathbf{s}$: $-J\mathbf{M}\cdot\mathbf{s}$. The spin-density to electric field response is governed by

$$\chi_{\alpha\beta} = \frac{\partial s_\alpha}{\partial E_\beta} \tag{1}$$

The standard symmetry analysis of response functions such as $\chi_{\alpha\beta}$ that depend on the orientation of a magnetic order parameter $\mathbf{m} = \mathbf{M}/|\mathbf{M}|$ starts with an expansion in powers of $\mathbf{m}$:

$$\chi_{\alpha\beta} = \chi^{(0)}_{\alpha\beta} + \chi^{(1)}_{\alpha\beta\gamma} m_\gamma + \chi^{(2)}_{\alpha\beta\gamma\delta} m_\gamma m_\delta + \chi^{(3)}_{\alpha\beta\gamma\delta\lambda} m_\gamma m_\delta m_\lambda + \cdots \tag{2}$$

Strictly speaking such an expansion is valid when $\mathbf{M}$ is small, or the influence of $\mathbf{M}$ on $\chi_{\alpha\beta}$ can be taken as a perturbation. Since the distinction between the spin-transfer torque (STT) and the field-like torque (FLT) in SOT experiments lies in their different parities under $\mathbf{m} \to -\mathbf{m}$, one can associate the former with $\chi^{(1)}$, $\chi^{(3)}$, $\chi^{(5)}$,… and the latter with $\chi^{(0)}$, $\chi^{(2)}$, $\chi^{(4)}$,… The symmetry-allowed components of the STT and FLT in terms of $\chi^{(n)}$ and their angular dependences can then be obtained if the symmetry at the FM/NM interface is known, using the method explained in [2]. For later convenience, the nonzero components of $\chi^{(0)}$, $\chi^{(1)}$, $\chi^{(2)}$, $\chi^{(3)}$ for the (0001) surface of Mn_3Sn, which has C_{3v} symmetry, assuming $\mathbf{m}$ and $\mathbf{E}$ to be in the *xy* plane and one mirror plane to be perpendicular to the *x* axis, are listed below:

$$
\begin{aligned}
\chi^{(0)}_{\alpha\beta}: \quad & \chi^{(0)}_{xy} = -\chi^{(0)}_{yx} \equiv -a_1 \\
\chi^{(1)}_{\alpha\beta\gamma}: \quad & \chi^{(1)}_{xxy} = \chi^{(1)}_{yxx} = \chi^{(1)}_{xyx} = -\chi^{(1)}_{yyy} \equiv -b_1 \\
& \chi^{(1)}_{zyy} = \chi^{(1)}_{zxx} \equiv b_2 \\
\chi^{(2)}_{\alpha\beta\gamma\delta}: \quad & \chi^{(2)}_{yxxx} = -\chi^{(2)}_{xyyy} \equiv -c_1 \\
& \chi^{(2)}_{xyxx} = -\chi^{(2)}_{yxyy} \equiv -c_2 \\
& \chi^{(2)}_{xxyx} = -\chi^{(2)}_{yyxy} \equiv -c_3 \\
& \chi^{(2)}_{xxxy} = -\chi^{(2)}_{yyyx} = c_1 + c_2 + c_3 \\
& \chi^{(2)}_{zyyx} = \chi^{(2)}_{zyxy} = \chi^{(2)}_{zxyy} = -\chi^{(2)}_{zxxx} \equiv -c_4 \\
\chi^{(3)}_{\alpha\beta\gamma\delta\lambda}: \quad & \chi^{(3)}_{zxxxx} = \chi^{(3)}_{zyyyy} \equiv d_1 \\
& \chi^{(3)}_{zxxyy} = \chi^{(3)}_{zyyxx} \equiv d_2 \\
& \chi^{(3)}_{zxyxy} = \chi^{(3)}_{zyxyx} \equiv d_3 \\
& \chi^{(3)}_{zxyyx} = \chi^{(3)}_{zyxxy} = d_1 - d_2 - d_3 \\
& \chi^{(3)}_{xxxxy} \equiv d_4 \\
& \chi^{(3)}_{xxxyx} \equiv d_5 \\
& \chi^{(3)}_{xxyxx} \equiv d_6 \\
& \chi^{(3)}_{xxyyy} \equiv d_7 \\
& \chi^{(3)}_{xyxxx} \equiv d_8 \\
& \chi^{(3)}_{xyxyy} = d_6 + d_7 - d_8 \\
& \chi^{(3)}_{xyyxy} = d_5 + d_7 - d_8 \\
& \chi^{(3)}_{xyyyx} = d_4 + d_7 - d_8 \\
& \chi^{(3)}_{yxxxx} = \frac{d_4 + d_5 + d_6 + 3d_7}{2} - d_8 \\
& \chi^{(3)}_{yxxyy} = \frac{-d_4 - d_5 + d_6 - d_7}{2} + d_8 \\
& \chi^{(3)}_{yxyxy} = \frac{-d_4 + d_5 - d_6 - d_7}{2} + d_8 \\
& \chi^{(3)}_{yxyyx} = \frac{d_4 - d_5 - d_6 - d_7}{2} + d_8 \\
& \chi^{(3)}_{yyxxy} = \frac{-d_4 + d_5 + d_6 - d_7}{2} \\
& \chi^{(3)}_{yyxyx} = \frac{d_4 - d_5 + d_6 - d_7}{2} \\
& \chi^{(3)}_{yyyxx} = \frac{d_4 + d_5 - d_6 - d_7}{2} \\
& \chi^{(3)}_{yyyyy} = -\frac{d_4 + d_5 + d_6 + d_7}{2}
\end{aligned} \tag{3}
$$

However, when the NM layer is replaced by another magnetic material, such as Mn_3Sn that has its own magnetic order parameter **N** (assumed to be a vector for now), χ

in principle depends on the orientation of its order parameter as well. In particular, the part of χ that changes sign under $\mathbf{n} \to -\mathbf{n}$, where $\mathbf{n} = \mathbf{N}/|\mathbf{N}|$, is defined as the *magnetic* spin Hall effect in [2], in the absence of the NiFe layer. When $\mathbf{n}$ and $\mathbf{m}$ are separately well defined, which is usually the case in bilayer samples used in SOT experiments, the STT and FLT acting on $\mathbf{m}$ can therefore be further separated into contributions due to SHE and MSHE depending on the parity of χ under $\mathbf{n} \to -\mathbf{n}$, according to e.g. Table S1.

	FLT	STT
SHE	$\chi(\mathbf{m}+, \mathbf{n}+)$	$\chi(\mathbf{m}-, \mathbf{n}+)$
MSHE	$\chi(\mathbf{m}+, \mathbf{n}-)$	$\chi(\mathbf{m}-, \mathbf{n}-)$

Table S1: Separation of the spin-transfer torque (STT) and field-like torque (FLT) into contributions due to the SHE and MSHE based on the parity of χ under the separate reversal of $\mathbf{m}$ and $\mathbf{n}$. $\mathbf{m}+$ ($\mathbf{m}-$) means even (odd) under $\mathbf{m} \to -\mathbf{m}$, etc.

Following the above idea, we can generalize Eq. (2) to an expansion in powers of both $\mathbf{n}$ and $\mathbf{m}$. Although the order parameter of Mn_3Sn is different from a single vector, when it is rotated in the kagome plane by coupling to an external magnetic field the rigid rotation of the spin structure is described by a single azimuthal angle φ, and one can define the in-plane vector $\mathbf{n}$ as $\mathbf{n} = \sin\varphi\, \hat{x} + \cos\varphi\, \hat{y}$, consistent with the definition of φ in the main text. Note that such a choice makes $\mathbf{n}$ rotate clockwise with increasing φ. More generally the expansion can be performed for a tensor order parameter, such as the octupolar order parameter of Mn_3Sn, which we do not discuss here. The expansion in $\mathbf{n}$ and $\mathbf{m}$ gives

$$\begin{aligned}
\chi_{\alpha\beta} &= \chi^{(0)}_{\alpha\beta} \\
&+ \chi^{(1),m}_{\alpha\beta\gamma} m_\gamma + \chi^{(1),n}_{\alpha\beta\gamma} n_\gamma \\
&+ \chi^{(2),mm}_{\alpha\beta\gamma\delta} m_\gamma m_\delta + \chi^{(2),mn}_{\alpha\beta\gamma\delta} m_\gamma n_\delta + \chi^{(2),nn}_{\alpha\beta\gamma\delta} n_\gamma n_\delta \\
&+ \chi^{(3),mmm}_{\alpha\beta\gamma\delta\lambda} m_\gamma m_\delta m_\lambda + \chi^{(3),mnn}_{\alpha\beta\gamma\delta\lambda} m_\gamma n_\delta n_\lambda + \chi^{(3),nmm}_{\alpha\beta\gamma\delta\lambda} n_\gamma m_\delta m_\lambda + \chi^{(3),nnn}_{\alpha\beta\gamma\delta\lambda} n_\gamma n_\delta n_\lambda \\
&+ \ldots
\end{aligned} \tag{4}$$

where the superscripts of $\chi^{(n)}$ together with the last n subscripts denote which Cartesian components of $\mathbf{n}$ and $\mathbf{m}$ are to be multiplied with $\chi^{(n)}$. One can then associate different $\chi^{(n)}$ with the STT and FLT of SHE and MSHE according to Table S1.

In the NiFe-Mn_3Sn system considered here, since NiFe is polycrystalline, the bilayer structure has the C_{3v} symmetry of the (0001) surface of single-crystal Mn_3Sn. The results in Eq. (3) can therefore be directly used to get the $\chi^{(n)}$ that do not have mixed $\mathbf{n}$

and **m** superscripts. For the mixed ones, one just needs to keep in mind that $\chi^{(n)}$ of the same order but with different superscripts, such as $\chi^{(3),mnn}$ and $\chi^{(3),mmn}$, are different. To reduce the number of parameters we further ignore $\chi^{(3),mmm}$ and $\chi^{(3),nnn}$ which are higher than 2nd order in **m** or **n**. Finally we assume that the azimuthal angle of **m** is opposite to that of **n**, in the sense that $m_x = -n_x = \sin\varphi,\ m_y = n_y = \cos\varphi$, to account for the opposite sense of rotation between the sublattice moments of Mn_3Sn and the external magnetic field. Such a choice means that when φ increases **m** rotates clockwise while **n** rotates counterclockwise. Assuming that the electric field **E** is along x so that $\delta s_\alpha = \chi_{\alpha x}E$ with $E = 1$ for brevity, we obtain

$$
\begin{aligned}
\delta\boldsymbol{s}(\boldsymbol{m}+,\boldsymbol{n}+) &= \tilde{c}_1\sin(2\varphi)\hat{x} + (a_1 + \tilde{c}_2\sin^2\varphi + \tilde{c}_3\cos^2\varphi)\hat{y} + \tilde{c}_4\cos(2\varphi)\hat{z} \\
\delta\boldsymbol{s}(\boldsymbol{m}-,\boldsymbol{n}+) &= (\tilde{b}_1 + \tilde{d}_1\sin^2\varphi + \tilde{d}_2\cos^2\varphi)\cos\varphi\hat{x} \\
&\quad +(\tilde{b}_1 + \tilde{d}_3\sin^2\varphi + \tilde{d}_4\cos^2\varphi)\sin\varphi\hat{y} \\
&\quad +(\tilde{b}_2 + \tilde{d}_5\sin^2\varphi + \tilde{d}_6\cos^2\varphi)\sin\varphi\hat{z} \\
\delta\boldsymbol{s}(\boldsymbol{m}+,\boldsymbol{n}-) &= (\tilde{b}'_1 + \tilde{d}'_1\sin^2\varphi + \tilde{d}'_2\cos^2\varphi)\cos\varphi\hat{x} \\
&\quad +(\tilde{b}'_1 + \tilde{d}'_3\sin^2\varphi + \tilde{d}'_4\cos^2\varphi)\sin\varphi\hat{y} \\
&\quad +(\tilde{b}'_2 + \tilde{d}'_5\sin^2\varphi + \tilde{d}'_6\cos^2\varphi)\sin\varphi\hat{z} \\
\delta\boldsymbol{s}(\boldsymbol{m}-,\boldsymbol{n}-) &= \tilde{c}_5\sin(2\varphi)\hat{x} + (\tilde{c}_6\sin^2\varphi + \tilde{c}_7\cos^2\varphi)\hat{y} + \tilde{c}_8\hat{z}
\end{aligned}
\tag{5}
$$

where the parameters are redefined to simplify the expressions, and we have used tilde and prime to distinguish them from those in Eq. (3). The expressions quoted in the main text have those marks removed for simplicity. Note that the expressions for the STT of SHE and the FLT of MSHE are identical, albeit the different parameters, and therefore cannot be separated in this symmetry approach. From Eq. (5) one can obtain τ^φ and τ^z of the STT/FLT due to SHE/MSHE respectively.

Note 5 Quantum kinetic theory of the spin-transfer torque in terms of the spin-electric-field response function

In this section we discuss a system consisting of two coupled layers. One of them (denoted by F) is a soft ferromagnet representing NiFe in a ST-FMR setup, and the other (denoted by S which stands for "source") is either a non-magnetic metal (such as Pt) or an antiferromagnetic metal (such as Mn_3Sn). Based on the symmetry analysis in the previous section, the essence of separating STT and FLT is whether the response function χ is even or odd under reversal of the magnetic order in F.

We first discuss how magnetization reversal affects response functions in the quantum kinetic theory framework. Denoting the time-reversal operator as $\mathcal{T}$, which is equal to $i\sigma_y\mathcal{K}$ for spin-1/2 fermions, one can generally partition any Hamiltonian into $\mathcal{T}$-even and $\mathcal{T}$-odd parts

$$H = \frac{1}{2}(H + \mathcal{T}H\mathcal{T}^{-1}) + \frac{1}{2}(H - \mathcal{T}H\mathcal{T}^{-1}) \equiv H^e + H^o. \tag{6}$$

If the $\mathcal{T}$-symmetry breaking in a mean-field-type Hamiltonian is due to a magnetic order parameter **M**, one must have

$$H^e(-\mathbf{M}) = H^e(\mathbf{M}), \quad H^o(-\mathbf{M}) = -H^o(\mathbf{M}). \tag{7}$$

The density matrix ρ can also be partitioned in the same way and satisfies similar relations to Eq. (7). Only the $\mathcal{T}$-odd part of ρ contributes to the expectation value of any $\mathcal{T}$-odd operator, and vice versa, because

$$\langle O \rangle = \mathrm{Tr}[(\rho^e + \rho^o)O] = \mathrm{Tr}[(\mathcal{T}\rho^e\mathcal{T}^{-1} - \mathcal{T}\rho^o\mathcal{T}^{-1})O] = \mathrm{Tr}[(\rho^e - \rho^o)\mathcal{T}O\mathcal{T}^{-1}]. \tag{8}$$

We now focus on the effect of $\mathcal{T}$ on the nonequilibrium density matrix from the quantum kinetic equation. Consider steady state only and start from the disorder-averaged kinetic equation:

$$\frac{i}{\hbar}[H_0, \delta\rho] + \frac{i}{\hbar}[H_E, \rho] + J[\delta\rho] = 0 \tag{9}$$

where $\delta\rho$ is the nonequilibrium part of the density matrix, H_0 is the unperturbed, disorder-free Hamiltonian, H_E is the perturbation due to an external electric field, and J is a functional describing the effect of disorder. We note that even in the simplest Born approximation the time-reversal property of $J[\delta\rho]$ depends on that of the time-dependent disorder potential. In the constant relaxation time approximation, the functional J is simply $1/\tau$. Although in principle τ can also have even and odd parts under $\mathcal{T}$, we keep the dominant part which is even in $\mathcal{T}$. By acting $\mathcal{T}$ and $\mathcal{T}^{-1}$ on both sides of Eq. (9) and considering the fact that H_E is $\mathcal{T}$-even, we obtain

$$\frac{i}{\hbar}[\mathcal{T}H_0\mathcal{T}^{-1},\mathcal{T}\delta\rho\mathcal{T}^{-1}]+\frac{i}{\hbar}[H_E,\mathcal{T}\rho\mathcal{T}^{-1}]-\frac{\mathcal{T}\delta\rho\mathcal{T}^{-1}}{\tau}=0 \tag{10}$$

which can be viewed as the same equation as Eq. (9) but for time-reversed H_0, ρ, $\delta\rho$, and $\tau\to-\tau$. Since $\mathcal{T}H_0(\mathbf{M})\mathcal{T}^{-1}=H_0(-\mathbf{M})$ and $\mathcal{T}\rho(\mathbf{M})\mathcal{T}^{-1}=\rho(-\mathbf{M})$, $\mathcal{T}\delta\rho\mathcal{T}^{-1}$ solved from Eq. (10) must satisfy $\mathcal{T}\delta\rho\mathcal{T}^{-1}=\delta\rho(-\mathbf{M},-\tau)$, where $\delta\rho(\mathbf{M},\tau)$ is the solution of Eq. (9) (under the constant relaxation time approximation). Consequently, in the large τ (or good metal) limit when only the $O(\tau^0)$ and $O(\tau^1)$ terms of $\delta\rho$ are retained, we have

$$\mathcal{T}\delta\rho^{(0)}(\mathbf{M})\mathcal{T}^{-1}=\delta\rho^{(0)}(-\mathbf{M}),\qquad \mathcal{T}\delta\rho^{(1)}(\mathbf{M})\mathcal{T}^{-1}=-\delta\rho^{(1)}(-\mathbf{M}). \tag{11}$$

Using Eq. (11) and $\mathcal{T}\mathbf{s}\mathcal{T}^{-1}=-\mathbf{s}$ we get, for the nonequilibrium spin density

$$\begin{aligned}\delta\langle\mathbf{s}\rangle^{(0)}(\mathbf{M})&=\mathrm{Tr}\left[\mathcal{T}\delta\rho^{(0)}(-\mathbf{M})\mathcal{T}^{-1}\mathbf{s}\right]=-\delta\langle\mathbf{s}\rangle^{(0)}(-\mathbf{M}),\\ \delta\langle\mathbf{s}\rangle^{(1)}(\mathbf{M})&=-\mathrm{Tr}\left[\mathcal{T}\delta\rho^{(1)}(-\mathbf{M})\mathcal{T}^{-1}\mathbf{s}\right]=\delta\langle\mathbf{s}\rangle^{(1)}(-\mathbf{M}).\end{aligned} \tag{12}$$

Namely, the $O(\tau^1)$ contribution to $\delta\langle\mathbf{s}\rangle$ or χ is even under magnetization reversal, while the $O(\tau^0)$ contribution to $\delta\langle\mathbf{s}\rangle$ or χ is odd under magnetization reversal. $\chi^{(1)}$ (superscript means power of τ, not to be confused with the notation in Eq. (2)) therefore includes the FLT of SHE and the STT of MSHE, while $\chi^{(0)}$ includes the STT of SHE and the FLT of MSHE. To separate MSHE and SHE in each term, however, one has to make further assumptions. For example, we could treat the exchange field term in the F layer as a perturbation, characterized by a dimensionless perturbation parameter λ_F and keep contributions up to $O(\lambda_F^1)$. This is justified by the standpoint that the F layer should ideally act as a probe and any dependence on its order parameter should be as simple as possible. With this approximation the four contributions to χ as defined in Table S1 can be calculated separately according to Table S2.

	FLT	STT
SHE	$O(\tau^1,\lambda_F^0)$	$O(\tau^0,\lambda_F^1)$
MSHE	$O(\tau^0,\lambda_F^0)$	$O(\tau^1,\lambda_F^1)$

Table S2: Separation of spin-transfer torque (STT) and field-like torque (FLT) due to the SHE and MSHE through the parity of χ, by taking the good-metal limit (large τ) and treating the exchange field term in the F layer as a perturbation.

We next calculate the different terms in Table S2 using the quantum kinetic theory. Assuming that the electric field is uniform across the thickness of the bilayer system, we can obtain

$$\partial_{\mathbf{E}}\delta\rho = -\frac{e}{\hbar}\mathcal{L}^{-1}[\partial_{\mathbf{k}}\rho] \tag{13}$$

where $\mathcal{L}$ is the Liouvillian of the whole system, including the exchange field term in F (denoted as H_F^λ):

$$\mathcal{L}[O] \equiv -\frac{i}{\hbar}[H_0 + H_F^\lambda, O] - \frac{O}{\tau} \tag{14}$$

$\mathcal{L}$ is a linear operator on the matrix (operator) O, and is therefore a "superoperator". Up to the first order in the perturbation H_F^λ we have

$$\mathcal{L}^{-1} \approx (\mathcal{L}^0)^{-1} - (\mathcal{L}^0)^{-1}\mathcal{L}^\lambda(\mathcal{L}^0)^{-1} \tag{15}$$

where $\mathcal{L}^\lambda[O] \equiv -\frac{i}{\hbar}[H_F^\lambda, O]$. The matrix elements of $\mathcal{L}^\lambda$ in the eigenstate representation of H_0 are

$$(L^\lambda)_{mn,pq} = -\frac{i}{\hbar}[(H_F^\lambda)_{mp}\delta_{qn} - (H_F^\lambda)_{qn}\delta_{mp}] \tag{16}$$

Using which one can obtain the mn matrix elements of the FLT and STT contributions to $\partial_{\mathbf{E}}\delta\rho$:

$$\begin{aligned} &\text{FLT:} \quad -\frac{e}{\hbar}\ell_{mn}(\partial_{\mathbf{k}}\rho)_{mn} \\ &\text{STT:} \quad -\frac{ie}{\hbar^2}\ell_{mn}[\ell_{pn}(H_F^\lambda)_{mp}(\partial_{\mathbf{k}}\rho)_{pn} - \ell_{mp}(H_F^\lambda)_{pn}(\partial_{\mathbf{k}}\rho)_{mp}] \end{aligned} \tag{17}$$

where $\ell_{mn} = i\hbar/(E_{mn} - i\hbar/\tau)$ with $E_{mn} \equiv \epsilon_{m\mathbf{k}} - \epsilon_{n\mathbf{k}}$, and p is a dummy index. One can then separate $O(\tau^0)$ and $O(\tau^1)$ terms to get SHE and MSHE contributions to $\partial_{\mathbf{E}}\delta\rho$:

$$\begin{aligned} &O(\tau^0, \lambda_F^0): \quad -ie\hbar\frac{f_{mn}}{(E_{mn})^2}\mathbf{v}_{mn} \\ &O(\tau^1, \lambda_F^0): \quad \frac{e\tau}{\hbar}(\partial_{\mathbf{k}}f_n)\delta_{mn} \\ &O(\tau^0, \lambda_F^1): \quad -ie\hbar\left[\frac{f_{np}}{E_{mn}E_{np}^2}(H_F^\lambda)_{mp}\mathbf{v}_{pn} + \frac{f_{mp}}{E_{mn}E_{mp}^2}(H_F^\lambda)_{pn}\mathbf{v}_{mp}\right] \\ &O(\tau^1, \lambda_F^1): \quad e\tau\left[2\delta_{mn}\frac{f_{mp}}{E_{mp}^2}\mathrm{Re}[(H_F^\lambda)_{mp}\mathbf{v}_{pm}] + \frac{1}{\hbar}\frac{\partial_{\mathbf{k}}f_{mn}}{E_{mn}}(H_F^\lambda)_{mn}\right] \end{aligned} \tag{18}$$

where $f_{mn} \equiv f_m - f_n$ and $f_m \equiv f(\epsilon_{m\mathbf{k}})$ is the Fermi-Dirac distribution function; $\mathbf{v}_{mn} \equiv \mathbf{v}_{mn}(\mathbf{k})$ is the inter-band matrix element of the velocity operator at momentum $\mathbf{k}$. Also we assume that the bands are nondegenerate, and hence the energy differences in the denominators are always nonzero. Eq. (18) are however not complete for the following reason: For insulators at zero temperature the solution of the quantum kinetic equation must preserve the idempotency of the density matrix, i.e., $(\rho + \delta\rho)^2 = (\rho + \delta\rho)$. If ignoring $(\delta\rho)^2$, this leads to the requirement

$$\rho \cdot \delta\rho + \delta\rho \cdot \rho = \delta\rho \tag{19}$$

In the $\tau \to \infty$ limit Eq. (19) should be used together with the kinetic equation to solve for $\delta\rho$ since the Liouvillian is rigorously speaking not invertible in this limit. The consequence of Eq. (19) is that the matrix elements of $\delta\rho$ are zero except those between cross-gap states [5], namely

$$\delta\rho = \rho \cdot \delta\rho \cdot (1-\rho) + (1-\rho) \cdot \delta\rho \cdot \rho \tag{20}$$

Applying Eq. (20) to the Fermi-sea terms in Eq. (18) will remove the 1st term in the $O(\tau^1, \lambda_F^1)$ contribution, and multiply the $O(\tau^0, \lambda_F^1)$ contribution by $f_m(1-f_n) + (1-f_m)f_n = f_m + f_n - 2f_m f_n$. (For metallic systems one must also remove any unphysical intra-band contributions at the Fermi surface that do not preserve the total number of electrons, but the present case does not have such contributions.) With these changes one can obtain the response function $\chi^{\alpha\beta} = \partial_{E^\beta}\mathrm{Tr}[\delta\rho s^\alpha]$ which has the following four contributions

$$\begin{aligned}
&\text{FLT (MSHE):} && e\hbar \frac{f_{mn}}{(E_{mn})^2} \mathrm{Im}(v_{mn}^{\beta} s_{nm}^{\alpha}) \\
&\text{FLT (SHE):} && \frac{e\tau}{\hbar} (\partial_{k^\beta} f_n) s_{nn}^{\alpha} \\
&\text{STT (MSHE):} && \frac{2e\tau}{\hbar} (\partial_{k^\beta} f_m) \frac{\mathrm{Re}[(H_F^\lambda)_{mn} s_{nm}^{\alpha}]}{E_{mn}} \\
&\text{STT (SHE):} && 2e\hbar \frac{f_{np}(f_m + f_n - 2f_m f_n)}{E_{mn}E_{np}^2} \mathrm{Im}[(H_F^\lambda)_{mp} v_{pn}^{\beta} s_{nm}^{\alpha}]
\end{aligned} \tag{21}$$

In the model calculation below we will use a simple $H_F^\lambda = -J\mathbf{m} \cdot \mathbf{s}_F$, where $\mathbf{m}$ is the direction of the uniform magnetization in the F layer, and $\mathbf{s}_F$ is the spin operator summed over all sites in the F layer, for the convenience of discussing the angular dependence of χ.

Note 6 Toy model calculation of the STT and FLT in NiFe/Mn$_3$Sn bilayer

We consider the following bilayer toy model similar to that used in [4] (Supplementary Fig. 4).

$$\begin{aligned} H_0 &= -\sum_{\langle im,jn\rangle\alpha} t_{mn} c^{\dagger}_{im\alpha} c_{jn\alpha} - \sum_{im\alpha} \mu_m c^{\dagger}_{im\alpha} c_{im\alpha} - J_S \sum_{i\alpha\beta, m<4} \hat{e}_m \cdot \boldsymbol{\sigma}_{\alpha\beta} c^{\dagger}_{im\alpha} c_{im\beta} \\ &+ \lambda_R \sum_{\langle im,jn\rangle\alpha\beta} i t_{mn} (\hat{z} \times \hat{d}_{im,jn}) \cdot \boldsymbol{\sigma}_{\alpha\beta} c^{\dagger}_{im\alpha} c_{jn\beta}, \end{aligned} \tag{22}$$

where i, j label unit cells; m, n label sublattices; α, β label spin. t_{mn} is the nearest-neighbor spin-independent hopping. $t_{mn} = 1$ is the energy unit if $m = n$, and $t_{mn} = t$ otherwise, standing for the inter-layer hopping. μ_m is a layer-dependent on-site potential: $\mu_m = \mu_S$ (μ_F) if $m < 4$ ($m \geq 4$). J_S is the on-site exchange field in the bottom (S) layer. $\hat{e}_m$ is the sublattice-dependent direction of the on-site exchange field. λ_R is the strength of a Rashba-type spin-orbit coupling due to inversion-symmetry breaking at the interface. $\hat{d}_{im,jn}$ is a unit vector pointing from site im to jn. The same scaling factor t as in the spin-independent hopping term is also included in the Rashba term to distinguish intra-layer and inter-layer hopping. Similar to [2], the main purposes of this toy model are to demonstrate the use of the formalism developed in the previous section and to qualitatively capture the symmetry-restricted STT and FLT responses due to SHE and MSHE measured in experiments. A typical band structure of the toy model is shown in Supplementary Fig. 5.

To calculate χ according to Eq. (21) we consider the following perturbation H_F^{λ}

$$H_F^{\lambda} = -J_F \hat{m} \cdot \sum_{i\alpha\beta, n\geq 4} \boldsymbol{\sigma}_{\alpha\beta} c^{\dagger}_{in\alpha} c_{in\beta} \tag{23}$$

The different contributions to $\delta\boldsymbol{s}$ or χ versus the azimuthal angle φ are plotted in Supplementary Fig. 6. One may notice that the $\delta\boldsymbol{s}$ corresponding to the FLT of MSHE and the STT of SHE is predominantly along z. Qualitatively speaking, this is because both of them originate from the inter-band contribution $O(\tau^0)$ to the nonequilibrium density matrix [2], and s_z has the largest inter-band matrix elements due to the form of spin-dependent terms in Eq. (22). Conversely, the $\delta\boldsymbol{s}$ corresponding to the STT of MSHE and the FLT of SHE is mostly in-plane, since the $O(\tau^1)$ contribution has to do with the intra-band matrix elements of spin. Such behaviors agree with the trend depicted in Fig. 5b of the main text.

From Supplementary Fig. 6 one can see that δs_z for the FLT of MSHE and STT of SHE has a dominant $\sin\varphi$ dependence. That the cancellation between the two contributions may make the higher-order angular dependence explicit mentioned in the

main text is exemplified in Supplementary Fig. 7. Since the STT of SHE depends on the value of J_F, we choose a $J_F = 0.357$ that allows the dominant $\sin\varphi$ dependence to be cancelled. The shape of $\delta s_z(\varphi)$ qualitatively agrees with that of $\tau^{\varphi}_{\mathrm{odd}}$ in Fig. 2h of the main text.

Supplementary Figure 8 shows an example that can be understood as the effect of inserting a Cu spacer discussed in the main text. Although our toy model cannot give the profile of $\delta\mathbf{s}$ along the thickness direction of the film, assuming the diffusive spin transport picture is approximately valid, the FLT will be more sensitive to the spacing between the S and F layers than the STT. Therefore inserting a Cu layer between NiFe and Mn_3Sn is expected to suppress the FLT more than the STT. As far as $\tau^{\varphi}_{\mathrm{odd}}$ or $\delta s^{z}_{\mathrm{odd}}$ is concerned, the effect of a Cu spacer can be simulated by increasing J_F, which makes the FLT of MSHE relatively decrease compared to the STT of SHE. The right panel of Supplementary Fig. 8 shows that with this change the $\delta s^{z}_{\mathrm{odd}}$ near $\varphi = 90^\circ$ can change its sign, qualitatively agreeing with the behavior in Fig. 4b of the main text.

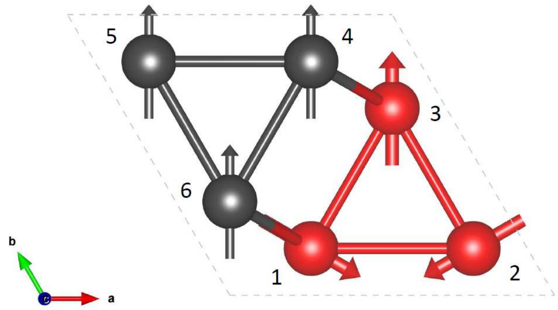

Supplementary Figure 4 Unit cell of the toy model in Eq. (22).

Red and gray balls represent the S and F layers, respectively. Red and gray arrows stand for the local moments in the S and F layers, respectively.

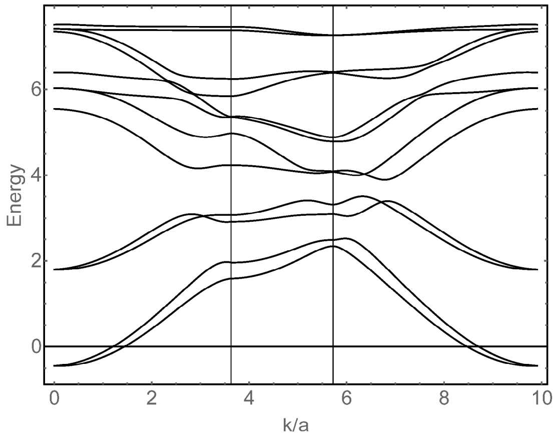

Supplementary Figure 5 Band structure of the toy model along the path $\boldsymbol{\Gamma-M-K-\Gamma}$. $J_S = 0.5,\ \lambda_R = 0.05,\ t = 0.5,\ \mu_S = -4.2,\ \mu_F = -5.2.$

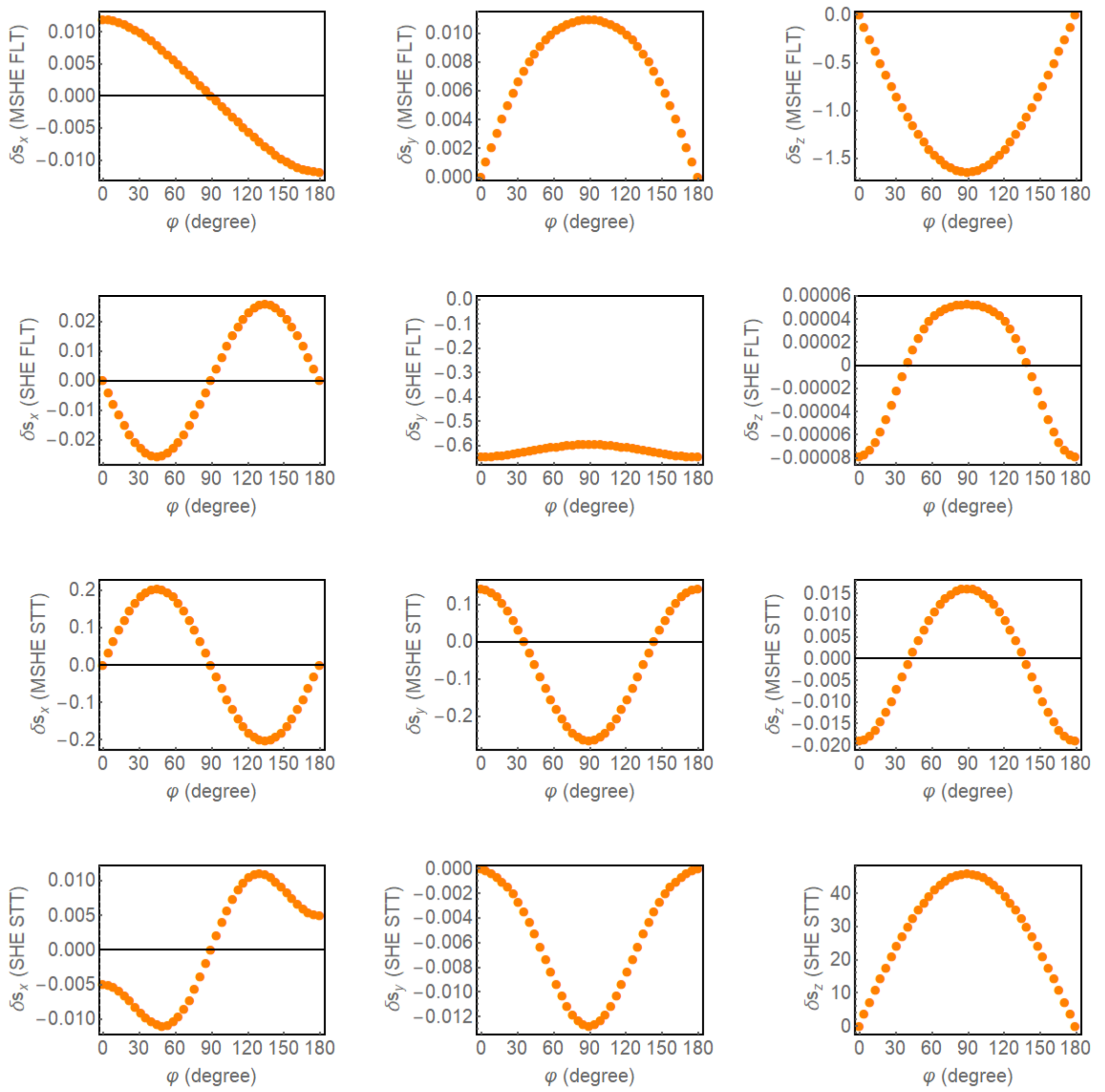

Supplementary Figure 6 Angular dependence of the different contributions to $\boldsymbol{\delta s} = \boldsymbol{\chi} \cdot \boldsymbol{E}$. $J_S = 0.5$, $\lambda_R = 0.05$, $t = 0.5$, $\mu_S = -4.2$, $\mu_F = -5.2$, and $k_B T = 0.05$. Spin is in units of $\hbar/2$. Both τ and J_F are set to 1. The electric field $\boldsymbol{E}$ is along x with unity magnitude. The k-space mesh used for the Brillouin zone integral is 141×141.

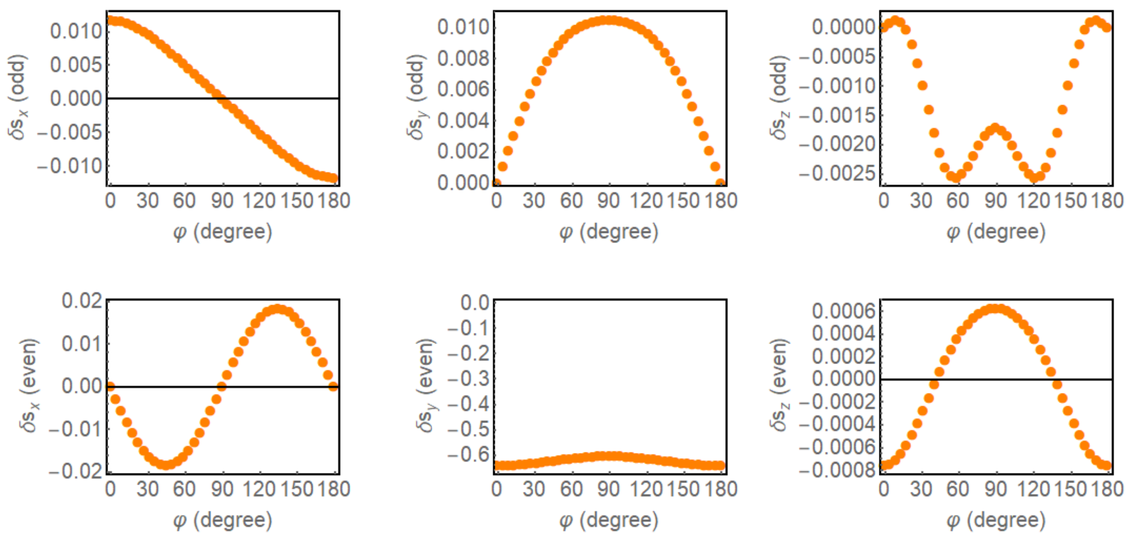

Supplementary Figure 7 Angular dependence of δs_{odd} (including the FLT of MSHE and STT of SHE) and δs_{even} (including the STT of MSHE and FLT of SHE), obtained from the data in Supplementary Fig. 6 but with $J_F = 0.0357$.

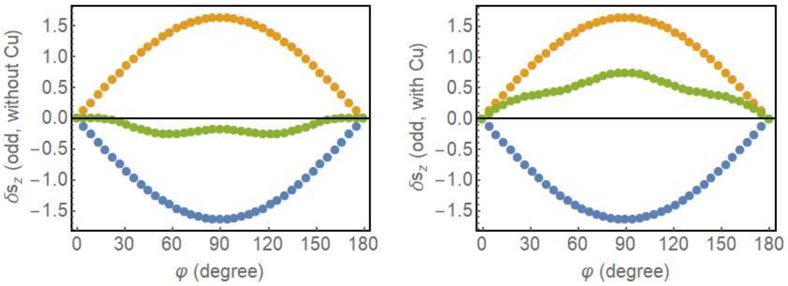

Supplementary Figure 8 Angular dependence of δs_{odd}^{z} without the Cu spacer (left) and with the Cu spacer (right). The orange, blue, and green dots represent the STT of SHE, FLT of MSHE, and their sum (enlarged by 100 times), respectively. The two panels are obtained from the same data as in Supplementary Fig. 6, but with $J_F = 0.0357$ (0.0359) in the left (right) panel.

Supplementary References

1. Liu, L., et al., Spin-torque ferromagnetic resonance induced by the spin Hall effect. PhysRevLett.106.036601 (2011).
2. M. Kimata et al. Nature 565, 627 (2019).
3. I. Garate and A. H. MacDonald, Phys. Rev. B 80, 134403 (2009).
4. F. Freimuth, S. Blügel, and Y. Mokrousov, Phys. Rev. B 92, 064415 (2015).
5. R. McWeeny, Phys. Rev. 126, 1028 (1962).
